# Hybrid Laparoscopic-Endoscopic Partial Jejunal Diversion with Magnetic Anastomosis in Obesity and Type 2 Diabetes: Durable 3-Year Metabolic Results

**DOI:** 10.1055/a-2892-7821

**Published:** 2026-06-23

**Authors:** Marek Buzga, Evžen Machytka, Marvin Ryou, Zdenek Svagera, Karolina Janochova, Jitka Machackova, Jan Kral, Veronika Horka, Martin Haluzik, Christopher C. Thompson

**Affiliations:** 1 Department of Physiology and Pathophysiology, Faculty of Medicine 48300 University of OstravaOstravaMoravian-Silesian RegionCzech Republic; 2 Institute of Laboratory MedicineFaculty of Medicine, University Hospital of OstravaOstravaMoravskoslezskýCzech Republic; 3 Department of HepatogastroenterologyInstitution for Clinical and Experimental MedicinePragueCzech Republic; 4 Division of Endocrinology, Diabetes and HypertensionBrigham and Women’s HospitalBostonMassachusettsUnited States; 5 Department of Gastroenterology 48228 University Hospital OstravaOstravaMoravian-Silesian RegionCzech Republic; 6 Department of Epidemiology, Faculty of Medicine 48300 University of OstravaOstravaMoravian-Silesian RegionCzech Republic; 7 Department of Clinical Subjects, Faculty of Medicine 48300 University of OstravaOstravaMoravian-Silesian RegionCzech Republic; 8 Department of Internal MedicineSecond Faculty of Medicine, Charles University, Motol and Homolka University HospitalPragueCzech Republic; 9 Department of Human Movement Studies, Faculty of Education 48300 University of OstravaOstravaMoravian-Silesian RegionCzech Republic; 10 Diabetes Centre 48214 Institute of Clinical and Experimental MedicinePraguePragueCzech Republic; 11 Division of Gastroenterology, Hepatology and EndoscopyBrigham and Women’s HospitalBostonMassachusettsUnited States

**Keywords:** endoscopy upper GI tract, foreign-bodies, endoscopy lower GI tract

## Abstract

**Background and Aims** Most patients with type 2 diabetes have overweight or obesity, yet few undergo bariatric surgery despite its superior metabolic efficacy over medical therapy. Less invasive alternatives are needed. We previously reported 12-month results of partial jejunal diversion (PJD) created using the incisionless magnetic anastomosis system (IMAS). We now report 36-month outcomes.

**Methods** This single-arm, prospective, first-in-human pilot study included 10 patients with obesity, including 4 with type 2 diabetes and 3 with prediabetes. A dual-path jejunal-ileal diversion was created using self-assembling magnets delivered endoscopically through colonoscopes, with laparoscopic port placement in all patients to confirm anastomosis position and assist magnetic coupling where required. After coupling, the magnetic device was expelled spontaneously. Endpoints included weight loss, glycated hemoglobin (HbA1c), and incretin metabolism.

**Results** PJD was successfully created in 10 consecutive patients (60% male; mean baseline BMI 41.1 kg/m ^2^ ). At 36 months, mean total body weight loss was 15.9% and excess weight loss was 43.2%. Mean HbA1c decreased by 1.8 percentage points in patients with diabetes and by 1.1 percentage points in those with prediabetes. No device-related serious adverse events occurred. Three serious procedure- or anatomy-related adverse events were observed: one trocar injury related to the laparoscopic component, one internal hernia requiring surgical repair, and one elective anastomosis reversal at patient request.

**Conclusions** Endoscopic PJD created using a self-assembling magnetic anastomosis system achieved durable weight loss and glycemic improvement through 36 months, with no device-related serious adverse events.

## Introduction

Between 2017 and 2018, population data from the National Health and Nutrition Examination Survey (NHANES) showed that the prevalence of obesity (body mass index (BMI) >30 kg/m ^2^ ) in the United States was 40.3% among younger men (aged 20–39), 46.4% among middle-aged men (aged 40–59), and 42.2% among older adults (aged 60 and over). Among women, the prevalence of obesity was 39.7% aged 20–39, 43.3% among those aged 40–59, and 43.3% among older women (aged 60 and over). 1 Obesity is a risk factor for type 2 diabetes, with NHANES data for 2019 showing that the prevalence of diabetes is 11.3%, and the prevalence of prediabetes 38.0%. 2 The majority of patients with type 2 diabetes have overweight or obesity; population-based estimates suggest that 85–90% of individuals with type 2 diabetes have a BMI ≥ 25 kg/m ^2^ . 3

Bariatric/metabolic surgery represents a highly effective treatment for obesity and type 2 diabetes compared to conventional medical interventions. 4 5 The results of a large number of animal and human studies indicate a key role for the digestive tract in the regulation of glucose homeostasis. 6 7 In addition to weight reduction, some types of surgery improve glycemic control independent of weight loss by affecting intestinal hormones, enterohepatic bile acid circulation, and composition of intestinal microbiota. 8 9 10 11 In many patients with diabetes, these changes reduce glycated hemoglobin (HbA1c) to nondiabetic levels and induce diabetes remission. Numerous studies show 30–63% remission rate of type 2 diabetes after surgery, with a follow-up of 1–5 years. 12 13 However, due to the invasiveness, risks, and costs of surgery, many patients with obesity and type 2 diabetes will not seek this type of treatment. It is estimated that out of all patients who meet the criteria for bariatric surgery, only ~1% of patients undergo surgery. 14

Bariatric endoscopy represents an emerging alternative solution. Many of these procedures use temporary implants. An endoscopic procedure that results in permanent anatomic alteration without residual foreign material would represent a paradigm shift. Our group has previously reported the development and application of the IMAS magnetic anastomosis system (GI Windows, Westwood, MA) for the creation of various types of gastrointestinal anastomoses. 15 These anastomoses were characterized by durability, large diameter, absence of permanent foreign material, and absence of leakage or bleeding.

The initial clinical application of the IMAS system focused on the endoscopic creation of a partial jejunal diversion (PJD), which has been explored as a surgical treatment for obesity and diabetes. 16 17 In this anatomy-preserving procedure, a side-to-side anastomosis (or dual-path intestinal diversion) is created between the proximal jejunum and the ileum such that a portion of nutrients and digestive fluid ingested circumvent most of the small intestine. However, preservation of the native path theoretically mitigates against the adverse effects of a blind, defunctionalized segment. Ultimately, the metabolic effects of PJD are expected to be similar to the hindgut mechanisms seen with biliopancreatic diversion with duodenal switch, single anastomosis duodenal-ileal bypass (SADI), or ileal transposition surgery.

In 2017, our group published interim 1-year results from a planned 3-year pilot study on the safety and efficacy of using the IMAS system to create PJD in 10 obese patients with T2DM. 18 The results of this study at 12 months showed a significant weight reduction, as well as an improvement in HbA1c in patients with diabetes and prediabetes. We now report data for 36 months on this patient cohort.

## Material and Methods

### Study Design and Patients

This was a single-arm prospective open-label first-in-human pilot study with a primary focus on subjects with obesity and type 2 diabetes or prediabetes designed to evaluate the safety, technical feasibility, and clinical performance (including metabolic effects) of IMAS when used to create a dual path intestinal diversion. The study was carried out at one site, the University Hospital of Ostrava, Ostrava, Czech Republic. The study was carried out with the approval of the Institution’s Ethics Committee and the State Institute for Drug Control, the regulatory authority of the Czech Republic. Informed consent was obtained from each study participant prior to enrolment (Clinical trial registration number: NCT02839512).

Inclusion criteria were age 18–65 years at screening and body mass index (BMI) 35–50 kg/m ^2^ . Those with a BMI between 35 and 40 kg/m ^2^ must have had at least one well-controlled clinically significant obesity-related comorbidity (e.g., type 2 diabetes, hypertension, dyslipidemia, sleep apnea). Key exclusion criteria were BMI > 50 or BMI < 35 kg/m ^2^ , type 1 diabetes, use of more than 2 oral antidiabetic medications, insulin, a dipeptidyl peptidase 4 inhibitor or a GLP-1 agonist; previous abdominal surgery; and hypersensitivity to nickel (the exoskeleton of the magnet device consists of a nickel–titanium alloy).

### Procedure and Assessments

With the patient under general anesthesia, pairs of self-assembling magnets (IMAS) were delivered by a deployment tool advanced through a colonoscope channel to the terminal ileum and the proximal jejunum, via simultaneous colonoscopy and enteroscopy. Laparoscopic ports were placed in each patient after magnet deployment to measure the exact position of the connection. Furthermore, a strict time limit of 40 minutes was imposed for the attempted magnetic coupling, as distention of the small intestine with a prolonged attempt could compromise laparoscopic assistance if necessary. In these cases, laparoscopic graspers were used to assist in coupling. The exact methodology of the procedure has been previously published. 15

Each procedure required simultaneous participation of three specialists: two endoscopists (one performing colonoscopy per anum for ileal IMAS deployment, one performing enteroscopy per os for jejunal IMAS deployment) and one laparoscopic surgeon providing visualization to confirm limb lengths and antimesenteric magnet positioning, and in 8 of 10 patients to assist magnetic coupling using laparoscopic graspers. Two separate endoscopic processing towers were required. A standard colonoscope was used for ileal magnet delivery via the per-anum route; the target zone of 50–100 cm proximal to the ileocecal valve was reached in all cases and confirmed by laparoscopic limb-length measurement. Fluoroscopic imaging was performed concurrently with laparoscopic visualization to confirm antimesenteric magnet positioning in real time. The term “incisionless” in the IMAS device name refers specifically to the creation of the anastomosis by magnetic compression necrosis, without mucosal incision, rather than to the overall procedure, which required laparoscopic port placement in all patients.

After device placement, patients were advised to consume a liquid/soft diet for the first two weeks, without specific dietary restrictions thereafter. No structured formal dietary or lifestyle modification program was mandated; this was an intentional study design element to evaluate the procedure’s intrinsic metabolic effect. Patients experiencing recurrent diarrhea received individualized nutritional counseling targeting reduction of simple carbohydrate intake. No caloric restriction, structured exercise program, or behavioral weight-loss intervention was prescribed. An abdominal X-ray was performed within 48 hours of the procedure to confirm the position of the magnets and was typically completed the day after the procedure according to the protocol. An upper GI series was performed 2 weeks after the procedure to confirm anastomotic patency and device passage. Follow-up endoscopies were performed 2, 6, and 12 months after device placement to confirm patency of the anastomosis.

The patients had follow-up clinic visits specific to the study in Months 1, 2, 3, 6, 9, 12, 18, 24, 30, and 36 after the original procedure. At each clinic visit, the subject was reviewed: medical history, adverse event evaluation, physical examination (including weight and girth measurements), and blood tests.

The primary endpoints were technical feasibility, defined as device deployment through the endoscope channel, successful IMAS engagement, patency of the anastomosis, and device-related serious adverse events. Secondary endpoints included percent total body weight loss (TBWL), percent excess weight loss (EWL), and change in HbA1c at 36 months. A mixed meal tolerance test (MMTT) was performed at baseline (before ingestion of mixed meal), which required preprandial and postprandial blood tests (−15 to 120 min) blood testing for glucose, insulin, C-peptide, glucagon, glucagon-like peptide 1 (GLP-1), YY peptide (PYY) and gastric inhibitory peptide (GIP) to evaluate the impact of the procedure on glycemic indices and intestinal hormones; these studies were repeated before and at months 6, 12, 18, 24 and 36 after the procedure.

### Hormonal and Biochemical Assays

Venipuncture was performed in the morning hours after fasting at night. Blood samples were processed for subsequent analysis 20 min after venipuncture. Serum glucose concentrations (AU 5420, Beckman Coulter, Inc., Brea, CA, USA) and HbA1c (Tosoh G8, Tosoh Bioscience, Japan) were measured using standard methods. Serum levels of gut hormones (ghrelin [active], glucose-dependent insulinotropic peptide [GIP, total], glucagon-like peptide 1 [GLP-1, active], pancreatic polypeptide [PP], and peptide YY [PYY]) were determined by a multiplex assay (MILLIPLEX MAP Human Gut Hormone Panel, Merck KGaA, Darmstadt, Germany) using barcoded magnetic beads and performed on a Bio-Plex MAGPIX instrument (BioRad, Hercules, CA, USA). Blood samples were kept at −80 °C until the time of analysis.

### Statistical Analysis

For all measured parameters, the degree of position (mean) and the degree of variability (standard deviation) were characterized. To verify the normality of the data distribution, we performed a Shapiro-Wilk test. A parametric independent t-test is used to assess the statistical significance of differences in means. The level of statistical significance was chosen for all tests at α = 0.05. A paired *t* -test (for continuous variables) or Wilcoxon test (for categorical data) was calculated to compare follow-up outcomes with baseline values to aid interpretation. Statistical processing was performed using IBM SPSS Statistics (Version 21; IBM, Armonk, NY, USA).

## Results

### Patient Demographics

A total of 14 patients were enrolled between October 2014 and March 2015, and 10 underwent successful IMAS placement. Three of the 14 patients were not tested (2 withdrew consent and the third was found to have lung disease, an exclusionary comorbidity). IMAS was not placed in the first 2 patients who had advanced to endoscopic evaluation due to the inability to approximate the appropriate bowel loops. This was thought to be due to intestinal distention due to air insufflation (instead of carbon dioxide) through colonoscopes during this initial evaluation. These patients did not experience adverse events. Subsequently, the procedure was modified to allow only carbon dioxide insufflation during the procedure. A total of 10 consecutive patients underwent an endoscopic evaluation and attempted IMAS placement. The demographic data are summarized in Table 1 . The study population was 60% male, had a mean age of 48.1 years (range: 22–58 years), a mean baseline BMI of 41.1 kg/m ^2^ (range: 34.7–46.2 kg/m ^2^ ). Four patients had type 2 diabetes, three had prediabetes (HbA1c 5.7%–6.4% and fasting glucose >100 mg/dL), and three did not have diabetes. Of the four patients with diabetes, three received oral medications and one was treated with diet alone.

**Table 1:** Summary of key patient demographics.

Variables	Value ± SD
Mean age [years]	48.1 ±10.5
Gender	
Male	6
Female	4
Mean daily caloric intake [kcal]	1896.3 ± 721.5
Mean weight at baseline [kg]	120.9 ± 17.8
Mean BMI [kg/m ^2^ ]	41.1 ± 4.3
T2DM/pre-diabetes/no diabetes	4/3/3
Mean age at diabetes onset [years]	43.0 ± 4.4
Mean baseline HbA1c [%]	
- 4 Diabetics	7.8 ± 2.4
- 3 Pre-diabetics	6.1 ± 0.3
Mean baseline fasting glucose [mg/dL]	
- 4 Diabetics	177.0 ± 93.0
- 3 Pre-diabetics	119.7 ± 7.3

### Technical Feasibility

**Fig. 1:**
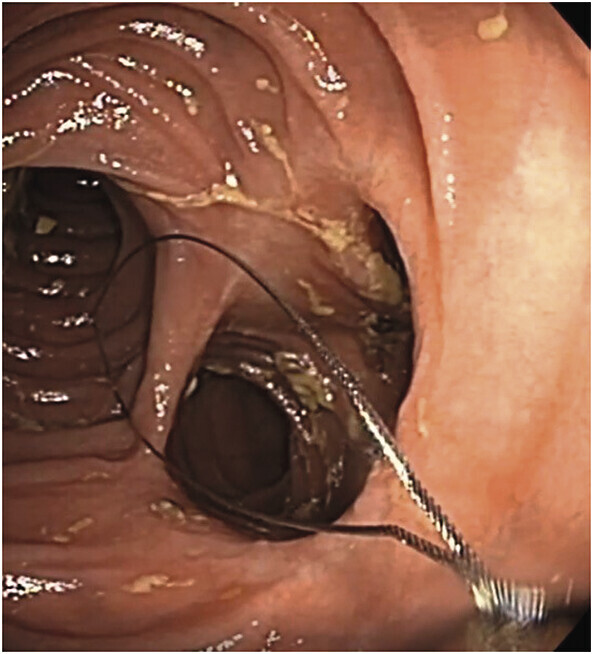
12-Month follow-up endoscopy with 25 mm snare in front of anastomosis.

Endoscopic delivery of IMAS to the desired segments of the intestine was successfully carried out through the colonoscope channel in all 10 patients. The target zone for the placement of the jejunal IMAS device was 50–100 cm distal to the Treitz ligament and the target zone for the placement of the ileal IMAS device was 50–100 cm proximal to the ileocecal valve. Laparoscopic visualization of the anastomosis site was also performed in all cases to confirm the length of the limb. Due to established time limits, laparoscopically assisted magnet coupling was used in the first 6 cases but was not required in 2 of the last 4 cases. Coupled magnets were confirmed to be in a fully antimesenteric position. The initial evaluation of the anastomoses was performed at 2 weeks through a series of upper GI with follow-up to the small intestine. A patent anastomosis ( Fig. 1 ) was observed in all patients, without evidence of leakage or perforation. Magnets were expelled on average 13 days after placement (range 8–28 days) in all but one patient.

### Clinical Outcomes

Durable weight loss was observed throughout 36 months, as depicted in Table 2 . The mean baseline weight of the study population was 120.9 ± 17.8 kg, which was reduced to 101.9 ± 22.2 kg at 36 months of follow-up ( *P* = 0.009). Progressive weight loss was observed in the first 12 months, which remained sustained during the total 36-month observation period. The mean EWL at 36 months was 43.2%. The mean TBWL at 36 months was 15.9%.

**Table 2:** Overview of the reduction in patient weight from baseline through month 36.

	Baseline	6 M	12 M	18 M	24 M	36 M
Weight [kg]	120.9 ± 17.8	108.0 ± 16.3	103.7 ± 21.5 ^†^	104.2 ± 23.4 ^†^	103.1 ± 22.9 ^†^	101.9 ± 22.2 ^†^
BMI [kg/m ^2^ ]	41.3 ± 4.4	36.9 ± 4.7 ^†^	35.4 ± 6.6 ^†^	35.6 ± 7.5 ^†^	35.2 ± 7.4 ^†^	34.8 ± 7.2 ^†^
Kg Lost [kg]	---	12.9 ± 7.7	17.3 ± 11.9 ^†^	16.8 ± 14.9	17.8 ± 15.1	19.1 ± 15.6
TBWL [%]	---	10.6 ± 5.9	14.6 ± 11.4	14.2 ± 13.7	15.0 ± 13.4	15.9 ± 13.7
EWL [%]	---	28.3 ± 17.3	40.2 ± 36.7	39.1 ± 42.4	41.9 ± 41.3	43.2 ± 41.8

^†^ Statistically significant difference vs. baseline ( *P* ≤ 0.05); comparison performed by Wilcoxon test.

BMI – Body Mass Index; TBWL – Total Body Weight Loss; EWL – Excess Weight Loss.

The mean baseline HbA1c of patients with diabetes was 7.8%, which was reduced to a mean of 5.9% at 36 months of follow-up, representing a mean change of −1.8% ( Table 3 ). In patients with prediabetes, the mean baseline was 6.1%, which decreased to a mean of 5.0% at 36 months (−1.1%). HbA1c decreased by 24.4% in patients with diabetes s and 18.0% in patients with prediabetes s.

**Table 3:** Overview of HbA1c and Fasting Glucose Reduction from baseline to month 36.

		Baseline	6 M	12 M	18 M	24 M	36 M
Fasting glucose[mg/dL]	DM	177.3 ± 93.0	110.0 ± 6.9	111.0 ± 10.0	123.0 ± 5.6	122.9 ± 30.3	123.6 ± 28.1
	Pre-DM	119.7 ± 7.3	104.4 ± 10.1	99.4 ± 5.5	98.0 ± 10.4	95.0 ± 10.4	95.3 ± 12.9
HbA1c [%DCCT]	DM	7.8 ± 2.4	6.0 ± 0.4 ^†^	5.9 ± 0.5 ^†^	6.6 ± 1.4 ^†^	5.9 ± 0.6 ^†^	5.9 ± 0.7 ^†^
	Pre-DM	6.1 ± 0.3	5.2 ± 0.3 ^†^	5.1 ± 0.2 ^†^	5.2 ± 0.2 ^†^	5.1 ± 0.3 ^†^	5.0 ± 0.2 ^†^

†Statistically significant difference vs. baseline ( *P* ≤ 0.05); comparison performed by Wilcoxon test.

The mean fasting glucose at baseline in patients with diabetes was 177 ± 93.0 mg/dL, which was reduced to a mean of 123.6 ± 28.1 mg/dL at 36 months of follow-up (decrease of 30.3%).

The MMTT was performed in 9 of 10 patients. The mean area under the curve (AUC) was determined for each analyte. The results between baseline and measurements at 6, 12, 18, and 24 months after the procedure were compared ( Table 4 ). The MMTT results show consistent and significant improvements in glucose tolerance across all measured timepoints. Simultaneously, there was a significant decrease in postprandial insulin levels. In terms of intestinal hormones, there was a numerically nonsignificant increase in PYY at some timepoints ( Table 4 ). There were statistically significant changes for GIP and GLP-1 at different timepoints. Ghrelin (active) showed a statistically significant progressive increase throughout the follow-up period from 12 months onward.

**Table 4:** Mixed-meal-tolerance testing from baseline to month 24*.

	AUC baseline, mean ± SD	AUC 6 month, mean ± SD	*p* Value (baseline to 6 month)	AUC 12 month, mean ± SD	*p* Value (baseline to 12 month)	AUC 18 month, mean ± SD	*p* Value (baseline to month 18)	AUC 24 month, mean ± SD	*p* Value (baseline to month 24)
Glucose [mg/dL]	1056 ± 465	807 ± 148	0.008 ^†^	854 ± 189	0.01 ^†^	922 ± 193	0.02 ^†^	927 ± 208	0.004 ^†^
C-peptide [pmol/L]	167,065 ± 43,607	131,398 ± 39,309	0.14	148,475 ± 65,668	0.65	141,025 ± 48,482	0.39	134,668 ± 51,595	0.2
Insulin [pmol/L]	76,579 ± 34,913	46,623 ± 35,311	0.008 ^†^	44,849 ± 45,463	0.027 ^†^	33,465 ± 32,493	0.044 ^†^	38,195 ± 24,784	0.022 ^†^
GLP-1 [pmol/L]	2399 ± 3312	1560 ± 678	0.86	1392 ± 598	0.027 ^†^	2190 ± 1333	0.32	2468 ± 2007	0.28
GIP [pmol/L]	7565± 3432	6259 ± 1987	0.21	6752 ± 2697	0.34	10543 ± 4997	0.05 ^†^	9681 ± 3216	0.1
PYY [pmol/L]	6838 ± 2799	7943 ± 1994	0.24	4635 ± 2809	0.7	6535 ± 2851	0.3	7026 ± 3302	0.13
Ghrelin (active) [pmol/L]	1855 ± 980	2007 ± 803	0.31	3301 ± 1617	0.001 ^†^	3170 ± 2022	0.001 ^†^	3296 ± 2220	0.021 ^†^

*Nine patients with obesity: 4 with type 2 diabetes mellitus, 2 with prediabetes, 3 did not have diabetes; 1 patient with prediabetes did not have a mixed meal tolerance test at baseline.

^†^ Statistically significant difference vs. baseline ( *P* ≤ 0.05); comparison performed by Wilcoxon test.

AUC – Area under the curve, GIP – gastric inhibitory peptide (glucose-dependent insulinotropic polypeptide), GLP-1 – glucagon-like peptide 1, PYY – peptide YY, SD – Standard deviation.

### Safety

Overall, the IMAS procedure was well tolerated. No device-related serious adverse events (SAEs) occurred. Three procedure- or anatomy-related SAEs were observed (30%): (1) an inadvertent trocar injury to the gastric serosa during laparoscopic port insertion (procedure-related; managed intraoperatively with suture repair, no sequelae); (2) surgical correction of an internal hernia at month 22 (anatomy-related; a recognized complication of any intestinal bypass anatomy, with a cumulative incidence of 2–6% after laparoscopic RYGB ^29^ ); and (3) elective surgical reversal of the anastomosis at month 31 at the patient’s explicit request due to persistent diet-related diarrhea in the context of a suboptimally placed anastomosis (<50 cm from the ileocecal valve) (anatomy-related, nonemergent). All three events were managed successfully without long-term adverse consequences.

**Fig. 2:**
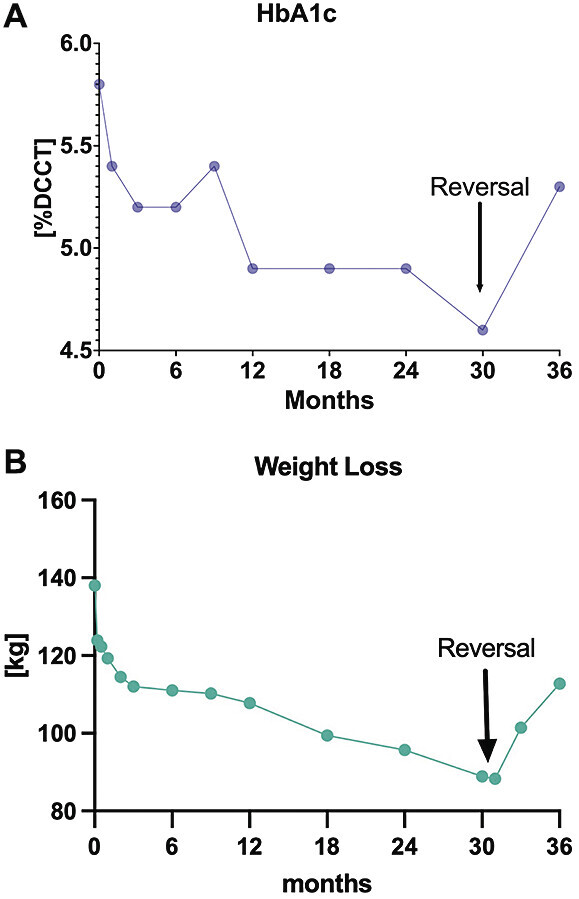
( **A** ) HbA1c increase after the patient had anastomosis reversal at 31 months postoperatively. ( **B** ) Weight gain after the patient had reversal of the anastomosis at 31 months postoperatively.

As shown in Table 5 , the procedure-related adverse events were primarily GI-related in nature and, at first, likely related to general anesthesia administration. Trocar site pain (coded as abdominal pain) was also experienced in the early postoperative period by all patients but resolved without intervention. All patients had diarrhea after the procedure in the short term. Recurrent diarrhea occurred in 4 patients (40%), and diarrhea in these patients appeared to be largely related to the composition of the diet. Dietary modification reducing simple carbohydrate intake resolved diarrhea in 3 of these 4 patients. However, one patient, whose anastomosis was located less than 50 cm from the ileocecal valve, continued to have diarrhea and elected surgical anastomosis reversal at month 31 using a linear stapler (anatomy-preserving reversal). Interestingly, the patient experienced a complete reversal of metabolic gains (weight regain of 27 kg and HbA1c increase of 0.8%) within 5 months, providing a compelling case-level demonstration of PJD’s metabolic mechanism ( Fig. 2A, 2B ). Additionally, another patient in month 22 underwent surgical correction of an internal hernia, a known complication of any intestinal bypass procedure.

**Table 5:** Adverse events over the 36 months.

Adverse events	Baseline	Month 1 *n* = 10	Month 6 *n* = 10	Month 12 *n* = 10	Month 18 *n* = 10	Month 24 *n* = 10	Month 36 *n* = 9
Gastrointestinal disorders							
Abdominal distension	0	1	0	1	1	0	0
Abdominal pain ^1^	0	2	0	1	2	0	0
Abdominal spasms	0	0	0	1	1	1	1
Bloating/Flatulency	0	4	2	2	3	2	2
Constipation	0	0	0	0	0	0	0
Diarrhea	0	8	7	5	5	3	4
Hemorrhoids	0	0	0	0	0	1	0
Nausea	0	1	0	0	0	0	0
Vomiting	0	1	0	0	0	1	0
Serious Adverse Events							
Ileus ^2^	0	0	0	0	0	1	0
Anastomosis reversed by surgery ^3^	0	0	0	0	0	0	1

^1^ Pain at the trocar site noted as abdominal pain.

^2^ Ileus diagnosed 22 months after PJD procedure.

^3^ Anastomosis reversed due to patient request 31 months after PJD procedure.

## Discussion

In this pilot study, we evaluated the technical feasibility, safety, and clinical results of IMAS when used to perform a PJD. We previously published an interim 12-month analysis that provided evidence of the effectiveness of IMAS in creating a durable intestinal diversion, while also documenting the safety profile and clinical efficacy of the procedure in patients with obesity, type 2 diabetes mellitus, and prediabetes. We now present positive evidence for the durability of these metabolic results up to 36 months.

All patients enrolled in the study experienced a significant weight reduction throughout the 36-month follow-up. The mean total weight loss in all patients was 19.1 kg, representing a TBWL of 15.9% with an EWL of 43.2%. Furthermore, a significant reduction in HbA1c and fasting glucose levels was observed at 36 months of follow-up. In patients with diabetes, the mean change in HbA1c was −1.8%. It is important to note that these durable metabolic changes were achieved in the absence of a formal diet and exercise program.

**Fig. 3:**
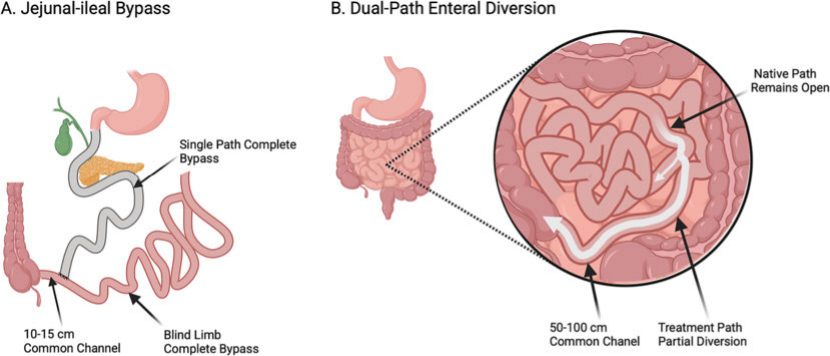
Jejunal-ileal bypass vs. dual-pathway diversion (Created in BioRender. Bužga, M. (2026) https://BioRender.com/y12t681 ) [rerif].

Unlike a traditional jejuno-ileal bypass, the anatomy created by IMAS is a PJD that preserves the native pathway for proper nutrient absorption and avoids a blind limb scenario ( Fig. 3 ). Our 12- and 36-month metabolic results are similar to those reported in other PJD studies created surgically. Fried et al. reported 15 patients with diabetes (12 on insulin) who achieved a TBWL of 10.3% and a change in HbA1c of −2.3% at 12 months. 19 Melissas et al. reported 6 patients with TBWL 11.9% and mean change in HbA1c of −2.0% at 36 months (3 patients [50%] with full diabetes remission). 20 In general, the improvement in glucose homeostasis after PJD using the IMAS device is comparable to the remission of diabetes observed after conventional bariatric surgery (RYGB and sleeve gastrectomy). 21 22

Gut hormones, including PYY, GIP, GLP-1, and ghrelin, were altered, suggesting a metabolic mechanism of action. The most consistent and clinically meaningful hormonal signals were the reductions in postprandial glucose AUC and insulin AUC, which were statistically significant at all measured timepoints and directly reflect improved glycemic control and reduced insulin demand — the primary metabolic goals of the procedure.

GLP-1 was significantly elevated only at the 12-month timepoint; PYY showed no significant change at any timepoint. This pattern differs from what is typically reported after biliopancreatic diversion or RYGB, where hindgut stimulation reliably and durably increases both GLP-1 and PYY. 23 24 Several factors may explain this heterogeneity: (1) the target range for anastomosis placement of 50–100 cm from the ileocecal valve introduces substantial variability in the volume of ileum receiving early nutrient contact, generating correspondingly heterogeneous hindgut stimulation; (2) the small sample ( *n* = 9 in MMTT) limits statistical power to detect moderate effects; (3) inter-patient variability in mixed-meal composition may contribute. We acknowledge that the heterogeneity of GLP-1 and PYY data is a limitation of this pilot cohort.

Active ghrelin increased significantly from 12 months onward. Elevated ghrelin following weight loss has been associated with a small but statistically significant compensatory drive toward weight regain in diet-induced weight loss cohorts, although the effect size is modest and the overall evidence for causal weight regain is inconsistent across studies. 25 Importantly, in our cohort, ghrelin rose despite sustained weight loss, suggesting that PJD’s metabolic benefit operates primarily through mechanisms other than ghrelin suppression, likely altered bile acid circulation, intestinal microbiota changes, and direct hindgut hormonal effects. This profile is mechanistically distinct from sleeve gastrectomy, where active ghrelin typically declines substantially. From this point of view, PJD could be useful for patients who do not eat or refuse surgical bariatric procedures.

Overall, the nature and severity of the adverse events observed in this study were relatively mild and consistent with altered anatomy. There were no unanticipated adverse events. In particular, nutritional diarrhea was expected and all 10 patients (100%) reported diarrhea after the procedure. This was self-limited in 6 patients; dietary modification resolved diarrhea in 3 of the remaining 4. Diarrhea at 40% recurrence constitutes a clinically significant side-effect that must be addressed through preprocedural dietary counseling and prospective monitoring. Ensuring anastomosis placement within the target zone of 50–100 cm from the ileocecal valve and dietary restriction of simple carbohydrates appear to be the critical modifiable factors. However, one patient whose anastomosis was located less than 50 cm from the ileocecal valve continued to have diarrhea with episodic dietary indiscretions and elected to opt for anatomy-preserving surgical reversal. This was accomplished technically using a linear stapler to close the anastomosis. Interestingly, the patient experienced a complete reversal of metabolic gains, demonstrating a case study of the metabolic effects of PJD. Additionally, another patient underwent surgical correction of an internal hernia at month 22, which is a known complication of intestinal bypass procedures. Its cumulative incidence after laparoscopic RYGB is approximately 2–6%, depending on mesenteric defect closure technique, and the single case observed in this 10-patient cohort is within the expected range for a small-sample bypass series. 26

Nutritional status remained largely preserved, with the exception of progressive vitamin B12 and vitamin D deficiency. A parallel 36-month nutritional follow-up of this cohort assessed a comprehensive micronutrient panel including vitamins A, E, B1–B12, D, folate, ferritin, albumin, and total protein. 27 Vitamins A, E, B1, B2, B6, folate, ferritin, albumin, and total protein remained within physiological limits throughout follow-up, comparing favorably with the high prevalence of deficiencies reported after malabsorptive bariatric surgery. Notably, however, serum vitamin B12 declined progressively, with deficiency (defined as <148 pmol/L) developing in 80% of patients by 36 months. 25(OH)D deficiency persisted at 24 and 36 months despite a transient improvement at 12 months. These findings underscore the need for prophylactic vitamin B12 supplementation from procedure initiation and regular micronutrient monitoring throughout follow-up, consistent with recommendations following other intestinal bypass procedures.

Broader adoption will require technical refinement toward a fully endoscopic approach. In its current form, PJD using IMAS requires a three-specialist team (two advanced endoscopists and a laparoscopic surgeon), two endoscopic processing towers, general anesthesia, and laparoscopic port placement. These resource requirements limit deployment to tertiary bariatric centers with integrated surgical and endoscopic expertise. The most important pathway to broader adoption lies in fully endoscopic coupling: in 2 of the last 4 patients in this series, laparoscopic assistance was not required for magnetic coupling. If this technical threshold can be consistently achieved through refined deployment tools and standardized insufflation protocols, the procedure would transition to a true endoscopic-only intervention, substantially reducing resource requirements and eliminating trocar-related risk. Prophylactic mesenteric defect closure at the time of anastomosis creation merits prospective evaluation to mitigate internal hernia risk.

The limitations of our study include the relatively small number of subjects enrolled, all of whom were recruited from a single center, and the fact that there is no control group. Small intestinal bacterial overgrowth (SIBO) was not systematically evaluated using breath testing. Although recurrent diarrhea resolved consistently with dietary intervention targeting simple carbohydrates rather than with antibiotic therapy, a contribution of SIBO cannot be excluded, particularly in patients with persistent symptoms; future studies should incorporate standardized hydrogen/methane breath testing at scheduled intervals. Validated health-related quality-of-life instruments (e.g., IWQOL-Lite, SF-36) were administered but achieved insufficient response rates for quantitative analysis; patient adherence to all 36-month study procedures provides indirect evidence of acceptable tolerability, and future studies should prioritize systematic validated QoL assessment. The variability in anastomosis placement location (50–100 cm from the ileocecal valve) may contribute to heterogeneity in hormonal and clinical outcomes and should be standardized in future protocols.

## Conclusion

In conclusion, this three-year study supports the technical feasibility and metabolic effectiveness of IMAS for creation of a durable PJD, while also highlighting clinically relevant procedure- and anatomy-related adverse events that require careful prospective management. PJD appears to have a beneficial effect on gut hormones that play a role in the pathophysiology of type 2 diabetes. Routine prophylactic vitamin B12 supplementation and regular micronutrient monitoring are recommended following PJD.

## References

[1] Hales C M Carroll M D Fryar C D Ogden C L Prevalence of obesity and severe obesity among adults: United States, 2017–2018 NCHS Data Brief 2020 360 1 8 32487284

[2] Prevention CfDCa National Diabetes Statistics Report 2026

[3] Maula A Kai J Woolley A K Educational weight loss interventions in obese and overweight adults with type 2 diabetes: a systematic review and meta-analysis of randomized controlled trials Diabet Med 2020 37 04 623 635 10.1111/dme.14193 31785118 PMC7154644

[4] Bhandari V Kosta S Bhandari M Bhandari M Mathur W Fobi M Bariatric metabolic surgery: an effective treatment of type 2 diabetes J Minim Access Surg 2022 18 03 396 400 10.4103/jmas.JMAS_325_20 34259204 PMC9306138

[5] Affinati A H Esfandiari N H Oral E A Kraftson A T Bariatric surgery in the treatment of type 2 diabetes Curr Diab Rep 2019 19 12 156 10.1007/s11892-019-1269-4 31802258 PMC7522929

[6] Kamvissi-Lorenz V Raffaelli M Bornstein S Mingrone G Role of the gut on glucose homeostasis: lesson learned from metabolic surgery Curr Atheroscler Rep 2017 19 02 9 10.1007/s11883-017-0642-5 28185153 PMC5306308

[7] Holst J J Gribble F Horowitz M Rayner C K Roles of the gut in glucose homeostasis Diabetes Care 2016 39 06 884 892 10.2337/dc16-0351 27222546

[8] Liu T Zou X Ruze R Xu Q Bariatric surgery: targeting pancreatic β cells to treat type II diabetes Front Endocrinol 2023 14 1.03161E6 10.3389/fendo.2023.1031610 PMC9975540 36875493

[9] Liou A P Paziuk M Luevano J M Jr Machineni S Turnbaugh P J Kaplan L M Conserved shifts in the gut microbiota due to gastric bypass reduce host weight and adiposity Sci Transl Med 2013 5 178 178ra41 10.1126/scitranslmed.3005687 PMC3652229 23536013

[10] Liu H Hu C Zhang X Jia W Role of gut microbiota, bile acids and their cross-talk in the effects of bariatric surgery on obesity and type 2 diabetes J Diabetes Investig 2018 9 01 13 20 10.1111/jdi.12687 PMC5754516 28434196

[11] Shetye B Hamilton F R Bays H E Bariatric surgery, gastrointestinal hormones, and the microbiome: an obesity medicine association (OMA) clinical practice statement (CPS) 2022 Obes Pillars 2022 2 100015 10.1016/j.obpill.2022.100015 37990718 PMC10661999

[12] Chumakova-Orin M Vanetta C Moris D P Guerron A D Diabetes remission after bariatric surgery World J Diabetes 2021 12 07 1093 1101 10.4239/wjd.v12.i7.1093 34326957 PMC8311476

[13] Mizera M Wysocki M Bartosiak K Type 2 diabetes remission 5 years after laparoscopic sleeve Gastrectomy: Multicenter cohort study Obes Surg 2021 31 03 980 986 10.1007/s11695-020-05088-w 33151518 PMC7920883

[14] Murtha J A Alagoz E Breuer C R Individual-level barriers to bariatric surgery from patient and provider perspectives: a qualitative study Am J Surg 2022 224(1 Pt B)429 436 10.1016/j.amjsurg.2021.12.022 34963509 PMC9218004

[15] Machytka E Buzga M Lautz D B Ryou M Simonson D Thompson C C 103 a dual-path enteral bypass procedure created by a novel Incisionless anastomosis system (IAS): 6-month clinical results Gastroenterology 2016 150 04 S26 10.1016/S0016-5085(16)30214-1

[16] Haluzík M Kratochvílová H Haluzíková D Mráz M Gut as an emerging organ for the treatment of diabetes: focus on mechanism of action of bariatric and endoscopic interventions J Endocrinol 2018 237 01 R1 r17 10.1530/joe-17-0438 29378901

[17] Ryou M Agoston A T Thompson C C Endoscopic intestinal bypass creation by using self-assembling magnets in a porcine model Gastrointest Endosc 2016 83 04 821 825 10.1016/j.gie.2015.10.023 26522371

[18] Machytka E Bužga M Zonca P Partial jejunal diversion using an incisionless magnetic anastomosis system: 1-year interim results in patients with obesity and diabetes Gastrointest Endosc 2017 86 05 904 912 10.1016/j.gie.2017.07.009 28716404

[19] Fried M Dolezalova K Chambers A P A novel approach to glycemic control in type 2 diabetes mellitus, partial jejunal diversion: pre-clinical to clinical pathway BMJ Open Diabetes Res Care 2017 5 01 e000431 10.1136/bmjdrc-2017-000431 PMC5706485 29225893

[20] Melissas J ErenTaskin H Peirasmakis D A simple food-diverting operation for type 2 diabetes treatment. Preliminary results in humans with BMI 28-32 kg/m(2) Obes Surg 2017 27 01 22 29 10.1007/s11695-016-2251-8 27581799

[21] Chang Y C Hsu C N Chong K Roux-en-Y and one-anastomosis gastric bypass surgery are superior to sleeve Gastrectomy in lowering glucose and cholesterol levels independent of weight loss: a propensity-score weighting analysis Obes Surg 2023 33 10 3035 3050 10.1007/s11695-023-06656-6 37612578

[22] Castellana M Procino F Biacchi E Roux-en-Y gastric bypass vs sleeve Gastrectomy for remission of type 2 diabetes J Clin Endocrinol Metab 2021 106 03 922 933 10.1210/clinem/dgaa737 33051679

[23] Miras A D Kamocka A Pérez-Pevida B The effect of standard versus longer intestinal bypass on GLP-1 regulation and glucose metabolism in patients with type 2 diabetes undergoing roux-en-Y gastric bypass: the long-limb study Diabetes Care 2021 44 05 1082 1090 10.2337/dc20-0762 33158945 PMC8132320

[24] McTigue K M Wellman R Nauman E Comparing the 5-year diabetes outcomes of sleeve Gastrectomy and gastric bypass: the National Patient-Centered Clinical Research Network (PCORNet) bariatric study JAMA Surg 2020 155 05 e200087 10.1001/jamasurg.2020.0087 32129809 PMC7057171

[25] Thom G McIntosh A Messow C M Weight loss-induced increase in fasting ghrelin concentration is a predictor of weight regain: evidence from the diabetes remission clinical trial (DiRECT) Diabetes Obes Metab 2021 23 03 711 719 10.1111/dom.14274 33289256

[26] Heck M Kensing B C Ismael H N Cecal volvulus as a rare complication of internal hernia after roux-en-Y gastric bypass: a case report and literature review J Surg Case Rep 2024 2024 04 rjae252 10.1093/jscr/rjae252 38666096 PMC11045239

[27] Bužga M Machytka E Švagera Z Macháčková J Janochová K Mizerová V Endoscopically created dual-path intestinal diversion using an incision-less anastomosis system in obese subjects: 3-year results of nutrition observation Physiol Res 2025 74(Suppl 1)S145 s54 10.33549/physiolres.935757 41511105 PMC12849770

